# Quantitative Assessment of Orbital Implant Position – A Proof of Concept

**DOI:** 10.1371/journal.pone.0150162

**Published:** 2016-03-03

**Authors:** Ruud Schreurs, Leander Dubois, Alfred G. Becking, Thomas J. J. Maal

**Affiliations:** 1 Department of Oral and Maxillofacial Surgery, Academic Medical Centre of Amsterdam (AMC) and Academic Centre for Dentistry (ACTA), University of Amsterdam, Amsterdam, the Netherlands; 2 3D Laboratory, Academic Medical Centre of Amsterdam (AMC) and Academic Centre for Dentistry (ACTA), University of Amsterdam, Amsterdam, the Netherlands; Georgia Regents University, College of Dental Medicine, UNITED STATES

## Abstract

**Introduction:**

In orbital reconstruction, the optimal location of a predefined implant can be planned preoperatively. Surgical results can be assessed intraoperatively or postoperatively. A novel method for quantifying orbital implant position is introduced. The method measures predictability of implant placement: transformation parameters between planned and resulting implant position are quantified.

**Methods:**

The method was tested on 3 human specimen heads. Computed Tomography scans were acquired at baseline with intact orbits (*t*0), after creation of the defect (*t*1) and postoperatively after reconstruction of the defect using a preformed implant (*t*2). Prior to reconstruction, the optimal implant position was planned on the *t*0 and *t*1 scans. Postoperatively, the planned and realized implant position were compared. The *t*0 and *t*2 scans were fused using iPlan software and the resulting implant was segmented in the fused *t*2 scan. An implant reference frame was created (Orbital Implant Positioning Frame); the planned implant was transformed to the reference position using an Iterative Closest Point approach. The segmentation of the resulting implant was also registered on the reference position, yielding rotational (pitch, yaw, roll) as well as translational parameters of implant position.

**Results:**

Measurement with the Orbital Implant Positioning Frame proved feasible on all three specimen. The positional outcome provided more thorough and accurate insight in resulting implant position than could be gathered from distance measurements alone. Observer-related errors were abolished from the process, since the method is largely automatic.

**Conclusion:**

A novel method of quantifying surgical outcome in orbital reconstructive surgery was presented. The presented Orbital Implant Positioning Frame assessed all parameters involved in implant displacement. The method proved to be viable on three human specimen heads. Clinically, the method could provide direct feedback intraoperatively and could improve postoperative evaluation of orbital reconstructive surgery.

## Introduction

The orbit is one of the most complex regions of the face in terms of reconstruction. Inaccurate restoration of orbital anatomy can result in functional dissimilarities and a poor aesthetical outcome. Unfortunately, sequellae of complex reconstructions are not rare [1,2]. Recent technological developments such as Computer Assisted Surgery (CAS), have proved to improve the safety and outcome of existing surgical procedures [3].

The use of the Computed Tomography scans for orbital reconstructive surgery has extended beyond diagnostic imaging alone [3]. By using advanced diagnostics software modalities such as iPlan (version 3.0.5; Brainlab^®^, Feldkirchen, Germany), the ideal location of the predefined implant, either preformed or patient-specific, can be assessed preoperatively. In navigation assisted surgery, the preoperative planning can be translated to the intraoperative setting and can provide the surgeon a guide map to the predetermined ideal implant location. The use of preoperative planning in combination with intraoperative navigation to aid the surgeon in reaching optimal surgical outcome is encapsulated in the surgical concept of CAS.

**Fig 1:**
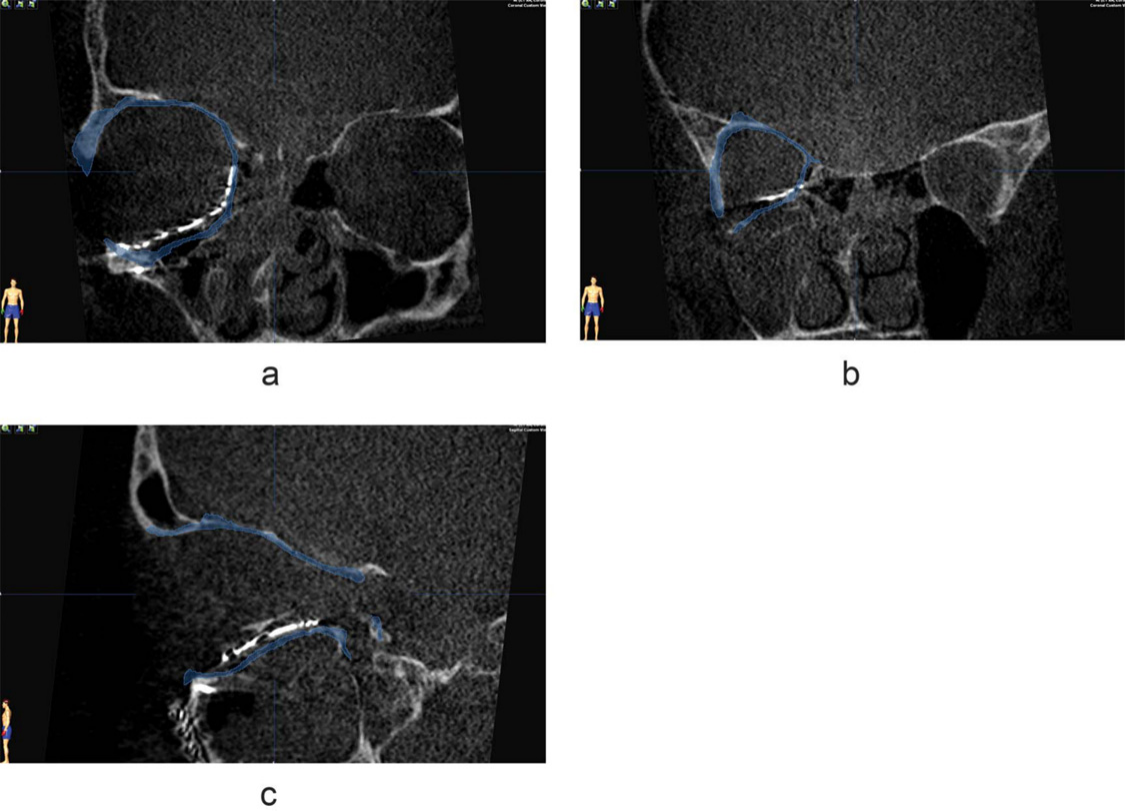
Resulting implant position of a preformed implant. Intraoperative imaging coronal views (a,b) and sagittal view (c) of the position of a preformed implant after orbital reconstruction. The mirror image of the unaffected orbit is visualized in blue. Based on the coronal and sagittal view, a good implant fit in relation to the mirrored orbit is seen.

**Fig 2:**
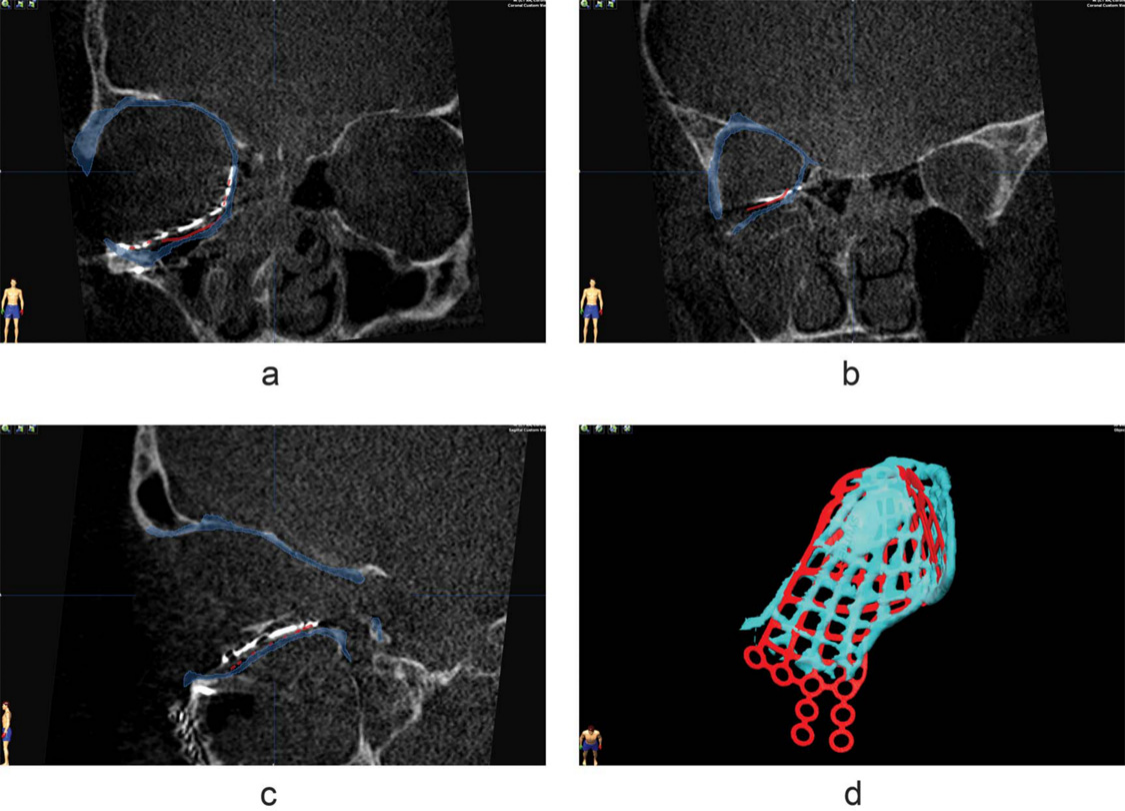
Resulting implant position compared to planned position of a preformed implant. Coronal views (a,b) and sagittal view (c) of the acquired implant position in relation to the planned implant position (red). Although the fit seems nice on the slices, there is a discrepancy between the planned implant position and the acquired position. In the three dimensional view (d), a rotation as well as translation of the final implant is seen; the target location has not been reached.

In orbital reconstructive surgery, CAS provides the surgeon a target location of the preformed implant to provide the best possible anatomical reconstruction of the affected orbit. Through imaging of the acquired position either intraoperatively or postoperatively, objective assessment of the surgical result in comparison to the target result is possible. In literature, various methods have been described to quantify implant location in patients, ranging from linear measurements on individual CT slices to color-coded distance maps [4–15]; the mirrored orbit is most often chosen as the target surface to which measurements of implant location are related [4,6–13]. In order to be able to objectively assess surgical accuracy, patient dependency should be excluded from the measurements. Local measurements on either one or multiple planar views alone will not be able to provide accurate information about translation and rotation of the implant to the surgeon. A clinical example is provided in Fig 1. Distance measurements on the coronal and sagittal slices visualized would yield small differences, some of which could be related to differences in the anatomical shape of the orbit in the patient and the shape of the preformed implant [16]. A good implant position seems to have been reached. Only if the acquired position is visualized in relation to the planned implant, the deviation of the optimal position (rotation and translation) from the planned implant position becomes apparent (Fig 2). This information will be valuable for evaluation of the postoperative result.

Since a target location is already provided in CAS, the actual implant location should be related to the planned implant location. The shape of the preformed orbital implant is known; if a reference frame is created, rotational and translational deviations can be quantified, providing a ‘true’ three-dimensional evaluation of implant position. Measures of rotation and translation will provide direct feedback to the surgeon on the necessary adjustments to obtain the ideal implant location. Patient dependency is eradicated from the equation, making comparison between patients and populations reconstructed with the same preformed implant possible. This study presents a largely automated position measurement method by means of a reference frame for the newly designed KLS preformed Orbital Implant (KLS Martin, Tuttlingen, Germany); this will hereafter be referred to as the Orbital Implant Positioning Frame (OIPF).

## Methods

### Reference frame

**Fig 3:**
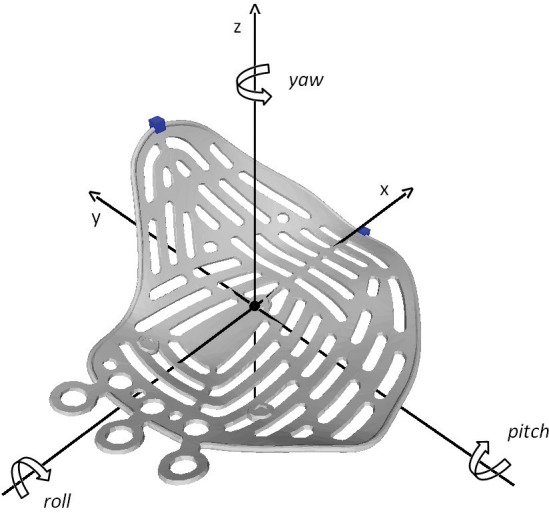
Orbital Implant Positioning Frame. The orientation of the axes, as well as the rotations around the axes are visualized. For the right-sided implant, the y-axis is flipped in order to be able to compare left and right sided implants. As a consequence of flipping the y-axis, the yaw and roll direction are also opposed.

The Orbital Implant Positioning Frame is visualized in Fig 3. The coordinate frame of the implant was designed bearing the principal axes of aircraft dynamics in mind [17]. The origin was pinpointed on the reinforced triangular part of the implant floor, intersected by both the longest axis of the maze and the most proximal contralateral bridges of the maze. The x-axis runs parallel to the longest axis to the tip of the implant, whereas the y-axis runs perpendicular to the x-axis and intersects the most proximal contralateral extensions; the perpendicular z-axis faces upwards. The rotations, which are expressed in pitch, yaw and roll, are also visualized in Fig 3.

### Human specimen study

Computer assisted orbital reconstructive surgery with a preformed titanium mesh plate was performed by LD on 3 human specimen heads with intact orbits, fixed in 2.4% formaldehyde. The specimen heads were supplied by the department of Anatomy of the Academic Medical Centre (AMC). Through a standard transconjunctival approach, complex orbital defects (Class III-IV) were created following the Jaquiéry classification [18,19], with Piezo surgery (Mectron, Carasco, Italy). CT scans (Sensation 64, Siemens Medical Solutions, Forchheim, Germany) of the human specimen heads were acquired at baseline (with intact orbits, *t*0), after creation of the orbital defects (*t*1) and postoperatively after implant placement (*t*2). The standard CT head trauma protocol was used (collimation 20 * 0.6 mm, with 120 kV, 350 mAs, pitch 0.85, FOV 30 cm, matrix size 512 * 512, reconstruction slice thickness 0.75 mm with overlapping increment 0.4 mm, in bone kernel H70s and bone window W1600 L400). The proposed method of measuring implant position described below was tested on the human specimen heads, in order to test the position measurement and demonstrate its feasibility.

### Planning

The *t*0 and *t*1 scans were fused using iPlan software; a 3D stereolithographic model (stl) of the preformed orbital implant was imported in the dataset. The stl of the preformed implant was positioned at the ideal location for anatomical reconstruction, based on information about the unaffected orbital contour (*t*0 scan). Information on existing bony ledges of the affected orbit (*t*1 scan), which is needed for adequate positioning of the implant, was also taken into account in finding the optimal fit. Consensus on the optimal implant position was reached between observers RS and LD for all planned implants.

### Segmentation

After the postoperative (*t*2) scan was acquired, the *t*0 and *t*2 scans were registered with the use of the Image Fusion modality in iPlan. A threshold-based segmentation with threshold ≥1200 HU was generated to segment the implant in the registered *t*2 scan; afterwards, post-processing of the segmentation result was performed by RS to remove superfluously segmented bony tissue. Care was taken not to include the three screw rings extending proximally from the implant, since these could have been adjusted during the surgical procedure; these bended areas of the implant could hamper surface registration between planned implant and the surgically positioned one. The fixation screw was also removed from the segmentation result.

### Implant placement evaluation

The planned implant was exported as an stl model (*M*_0_) from the iPlan software; the segmentation result was also exported in stl format (*M*_2_). The local coordinate frame of the planned implant was transformed to the global reference frame by matching the planned implant on the reference implant, with a best-fit matching approach (Iterative Closest Point algorithm) [20]:

$$M_{0,G}={\text{T}}_{L\to G}M_{0}$$ (1)

The same transformation (T_*L*→*G*_) was applied to the segmented implant, thereby maintaining the relative orientation between *M*_0_ and *M*_2_, but transforming them both to the global coordinate frame:

$$M_{2,G}={\text{T}}_{L\to G}M_{2}$$ (2)

The Iterative Closest Point (ICP) algorithm was also utilized to register the planned (*M*_0,*G*_) implant on the resulting (*M*_2,*G*_) implant, in order to determine the transformation matrix between the planned and acquired implant position:

$$M_{2,G}={\text{T}}_{0\to 2}M_{0,G}$$ (3)

From the resulting transformation matrix T_0→2_, the rotation parameters pitch (rotation around y-axis, *β*), yaw (rotation around z-axis, *α*) and roll (rotation around x-axis, *γ*) were calculated using the following formula:

$$R_{0\to 2}\left(\alpha ,\beta ,\gamma \right)=R_{z}\left(\alpha \right)R_{y}\left(\beta \right)R_{x}\left(\gamma \right)=\left(\begin{array}{ccc} \cos\alpha \cos\beta & \cos\alpha \sin\beta \sin\gamma -\sin\alpha \cos\gamma & \cos\alpha \sin\beta \cos\gamma +\sin\alpha \sin\gamma \\ \sin\alpha \cos\beta & \sin\alpha \sin\beta \sin\gamma +\cos\alpha \cos\gamma & \sin\alpha \sin\beta \cos\gamma -\cos\alpha \sin\gamma \\ -\sin\beta & \cos\beta \sin\gamma & \cos\beta \cos\gamma \end{array}\right)$$ (4)

The order of rotations in the resulting calculation was, by definition of the formula above, regarded as roll first, pitch second, and yaw third (respectively rotations around x-axis, around y-axis, around z-axis). The translational parameters of the origin in all three directions were also acquired from the transformation matrix T_0→2_. Since transformation of the complete implant was described by the transformation matrix, the translation of other key points on the implant (superiormost point on medial ledge, dorsal tip of implant) could be calculated if desirable. These points are also indicated in Fig 3. Since all planned implants were registered on the reference implant, rotations and translations could be compared for all surgical interventions. For the right-sided implant, the reference frame was mirrored over the *xz*-plane, in order to be able to compare results for left-sided and right-sided orbital reconstructions.

## Results

### Specimen 1

**Fig 4:**
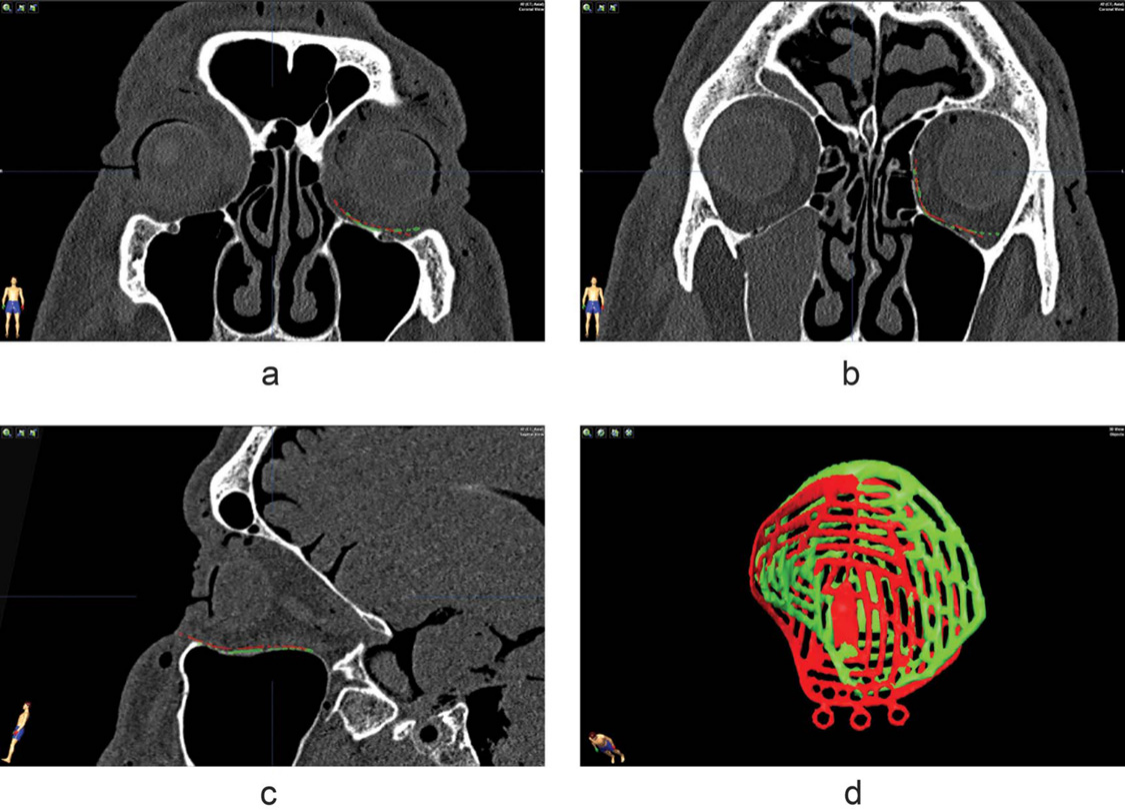
Implant position in specimen 1. Planned implant position (red) vs. final implant position (green) on the *t*0 scan of specimen 1. The slices (a,b,c) show a proper implant position, but in the 3D view a significantly different position of the resulting implant is seen compared to the planned implant position.

**Fig 5:**
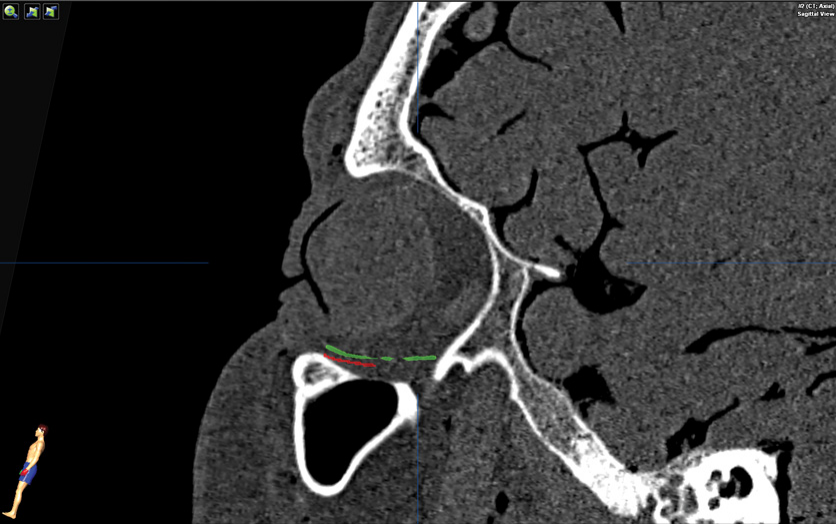
Lateral extension of the final implant in specimen 1. The planned implant is visualized in red, the final implant is visualized in green. Because of the rotation and translation of the implant compared to its planned position, interference of the lateral part of the implant with the inferior orbital fissure is present.

In Fig 4, the planned implant position and segmentation of the resulting implant position are visualized in red and green respectively, in both coronal (*a*,*b*) and sagittal view (*c*) of the baseline (*t*0) scan. In both coronal views shown here, as well as the sagittal view, comparison to the implant floor would suggest an adequate position of the resulting implant. In Fig 4B the lateral extension of the implant is positioned too far laterally, but overall the contour is nicely followed in these slices. A 3D view can be generated after the segmentation process described above; when the 3D view is taken into account, the resulting implant position turns out to significantly deviate from the planned implant position. Analysis with the method described above yields the following dislocation parameters: roll -9.3°, pitch -0.4°, yaw 11.3°, translation_x_ 2.5mm, translation_y_ -2.7mm, translation_z_ -0.5mm. A yaw combined with a lateral translation is present, and the target set by the planned implant position is obviously not reached. These translations and rotations will affect implant position at the inferior orbital fissure, as seen in Fig 5.

### Specimen 2

**Fig 6:**
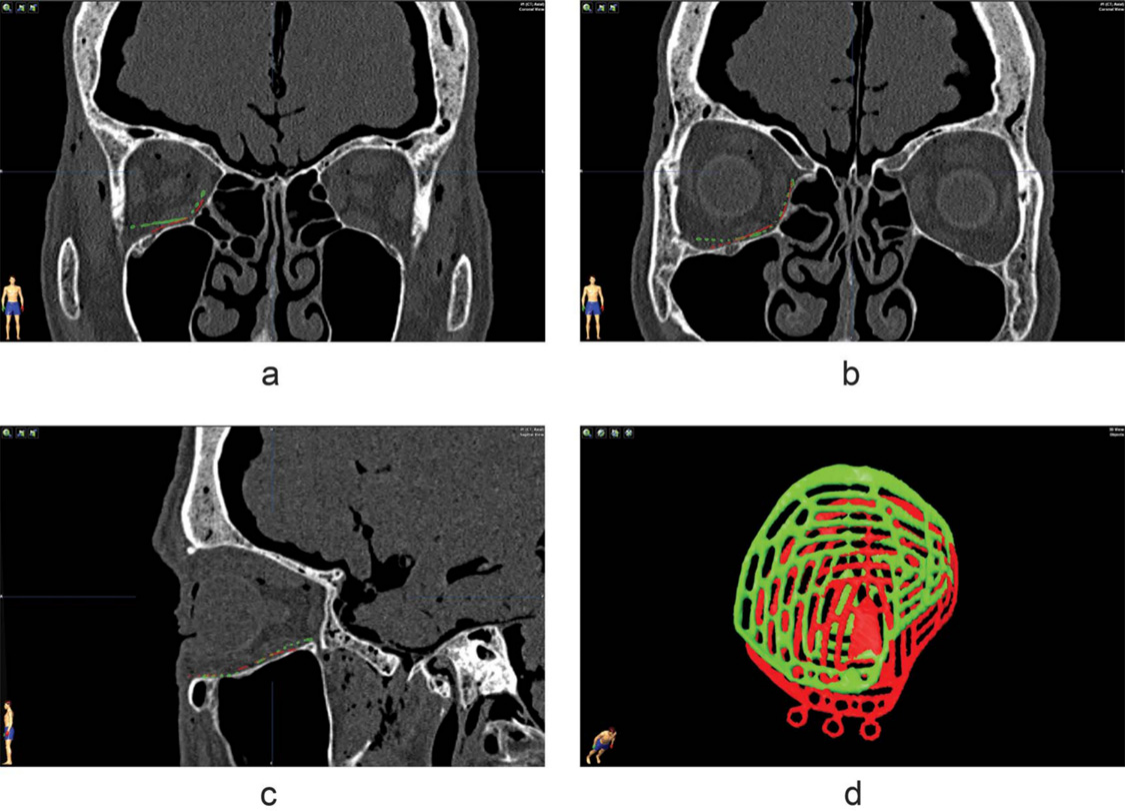
Implant position in specimen 2. Planned implant position (red) vs. final implant position (green) on the t0 scan of specimen 2. Based on the sagittal slice, a dorsal translation is present in the final implant position. The 3D view reveals that the implant is also translated laterally, this cannot be identified on the coronal and sagittal views alone.

**Fig 7:**
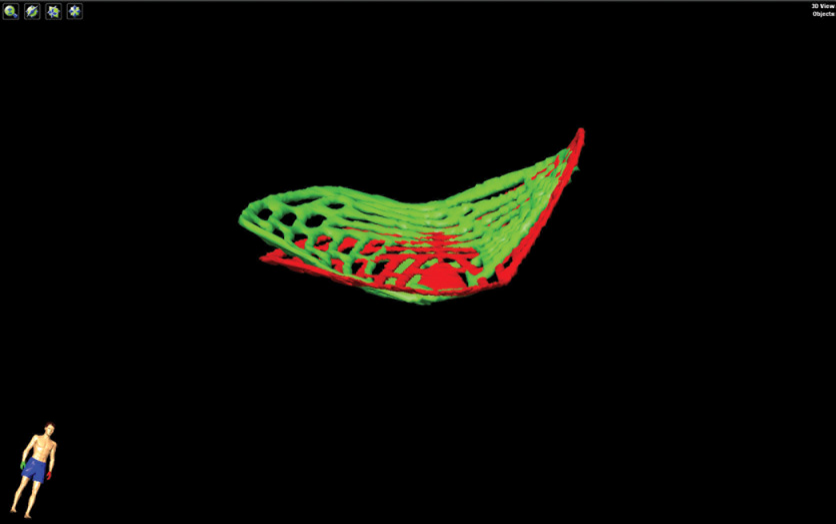
3D view of the planned and final implant position in specimen 2. The 3D view after segmentation of the implant illustrates that a roll is present: the resulting implant is rotated in a counterclockwise fashion in this view.

Fig 6 visualizes the implant placement in *specimen 2*. In the coronal slices, the orbital contour is nicely followed medially; laterally, there is a significant deviation between implant position and orbital floor. From the sagittal view it could be concluded that the implant is placed too far dorsal. In the coronal views, the cross-section through the resulting implant would be visualized at another level than the cross-section through the planned implant. This might explain the observed overlap between the planned and the resulting implant position medially, but with a lateral deviation. The three dimensional view, generated after segmentation, shows that a dorsal translation of the resulting implant is not the only translational error compared to the planned position; a lateral displacement is also present. Analysis with the Orbital Implant Positioning Frame shows that there are also a roll rotation in the resulting implant (roll -11.2°, pitch -5.3°, yaw 0.1°, translation_x_ 4.1mm, translation_y_ -2.9mm, translation_z_ -0.1mm); although this could not be concluded from the individual slices, it is clearly shown in the 3D view in Fig 7.

### Specimen 3

**Fig 8:**
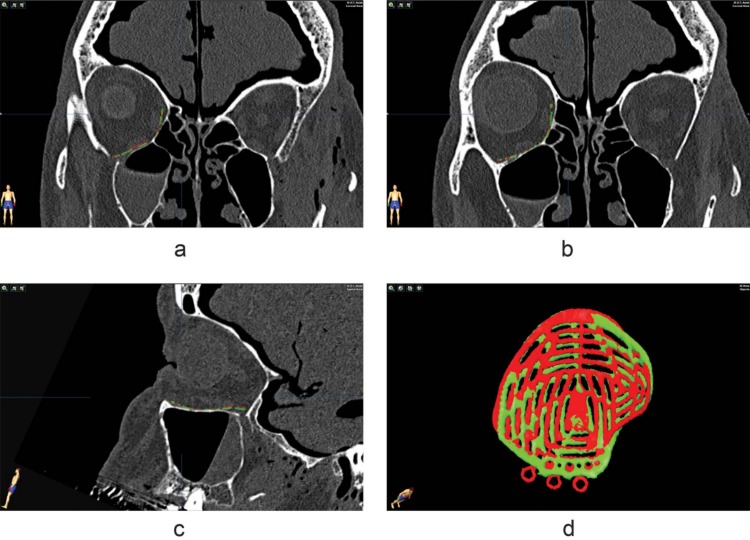
Implant position in specimen 3. Planned implant position (red) vs. final implant position (green) on the t0 scan of specimen 3. The relation of the final implant with both the planned implant and the orbital contour seems excellent; the 3D view shows the surgical target has indeed been reached, although a minor yaw seems to be present.

The implant positioned in *specimen 3* is in close relation to the planned implant position. On all slices visualized in Fig 8, only slight differences between planned implant and positioned implant are present; the contour of the orbit is nicely followed. The 3D view of the implants confirms that the planned implant position has been acquired during surgery. The parameters from the analysis are as follows: roll -1.1°, pitch 1.9°, yaw -7.1°, translation_x_ -0.3mm, translation_y_ 0.2mm, translation_z_ -0.4mm. In comparison to the parameters found in *specimen 1* and *specimen 2*, the dislocation parameters to the planned position are very small.

## Discussion

The goal of orbital implant placement is to reconstruct the bony orbit and to restore the orbital volume. Although Andrades et al. showed no direct correlation between implant position, orbital volume and clinical outcome [21], in more complex fractures (Jaquiéry Class III-IV) surgical outcome may be poor due to suboptimal positioning of the orbital implant [1]. Adverse clinical outcome of globe position like enophthalmos and hypoglobus is influenced by both orbital volume and the shape of the restored orbital contour, which are influenced by the position of the implant within the orbit. In the planning phase of orbital reconstruction, a digital model of the orbital implant can be imported in the planning environment. The implant position can be assessed without the disturbing protruding orbital soft-tissues and the optimal implant fit to recreate the anatomical shape of the orbit can be planned. In the clinical setting, it is important that the planned position of the implant does not interfere with the unaffected bony ledges of the affected orbit, because this would hamper placement of the implant on the planned position. The second factor in planning the implant position is the information provided by a mirrored segmentation of the hard-tissue contralateral side; thirdly, clinical considerations (i.e. a need for overcorrection in specific cases) could play a role in finding the optimal position in the planning process.

The planned position assuring optimal fit of the implant serves as the target in positioning of the implant during surgery. Image fusion of preoperative and postoperative scans provides the possibility to compare surgical result to the planning, to see if the target has been reached. A novel method for standardized position analysis of the acquired implant position is described in this article. This standardized position analysis compares the acquired position to the target position and is able to provide feedback to the surgeon about all implant dislocation parameters. A specimen study has been conducted to demonstrate the feasibility of the method to measure implant position; it would be of clinical interest to relate the dislocation parameters to clinical outcome such as resulting orbital volume, globe position and functional or esthetic deficits in an in-vivo study. From this clinical study it would be possible to establish an evidence-based range in which the difference between planned and acquired position is acceptable.

In complex defects (Class III-VI) contour becomes a more important factor for repositioning the globe in a proper position [1,18]. Patient-specific implants (PSI) are regarded to be perfectly shaped, and may hold the best potential for optimal restoration of the contour of the orbit [22]. Serious drawbacks of the use of a PSI for orbital reconstruction are relative high cost and limited availability through logistic factors. Various studies showed the potential of preformed implants as an easily usable and cost-effective alternative to PSIs for true-to-original orbital reconstruction [5,9,21]. The predefined shape makes the implant perfectly suitable for the concept of CAS, where the optimal implant position in relation to desired orbital contour can be planned beforehand.

**Fig 9:**
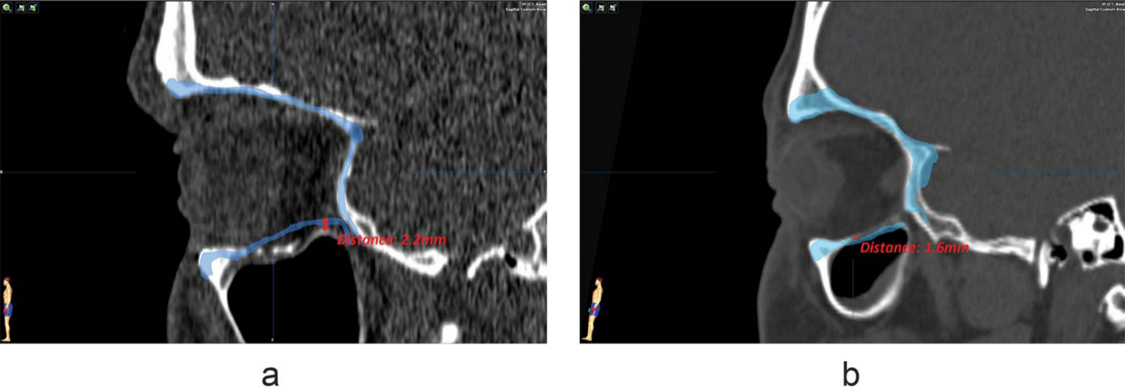
Differences in orbital symmetry. The mirror image of the unaffected orbit is visualized in blue. Distances are measured between unaffected bony parts of the affected orbit and the mirrored orbit. If the mirrored orbit would be used as a reference for implant position, the measurement in (a) would yield negative values at the posterior ledge; the difference in symmetry measured in (b) would add up to the dislocation measurement, since the implant would always be positioned cranial to existing bony structures.

In this study, a reference frame for a preformed implant has been designed. Rotation and translation between planned position and the segmented implant in the matched postoperative scan are calculated. In literature, various methods exist to assess implant position after surgery. Linear measurements between mirrored (unaffected) orbit and the positioned implant, on either one slice of the postoperative CT scan [4,5] or multiple slices [6,7,9–11], are frequently reported as a measure to quantify the acquired implant location in comparison to the ideal position. These measurements are reported to be hampered by the assumption of symmetry between affected and unaffected orbit and errors originating from the choice of measurement points [23]. A difference between bony ledges of the affected orbital floor and the mirrored unaffected orbit is visualized in Fig 9. If linear measurements are performed in this example, the difference in symmetry will affect the outcome of the implant dislocation measurement. Slice thickness and implant scatter are scan-related factors affecting accuracy of linear measurements. Reproducibility and accuracy of the measurements may be hampered by the mirroring process, since positioning of the mirrored orbit is performed manually.

If (color-coded) distance maps are used for quantification of the acquired implant position [8,12,13], errors originating from the selection of the measurement points are eradicated. Reproducibility and accuracy of the measurement may still be affected through mirroring. Using the mirrored orbit of the patient as the reference for quantifying surgical result has an additional disadvantage: it introduces inter-patient variability. Different orbital shapes yield different results, even if the planned position assuring optimal implant fit in the patient is acquired during surgery. The goal of this study is to present a method to evaluate implant placement in a way that comparison between different methods, surgeons and patients is feasible. Since the planned position sets the target for implant positioning during surgery, the planned position should be used to evaluate if the surgical target has been reached and the reconstruction has been performed according to plan.

Stoor et al. did choose the planned implant as the reference, and calculated the volumetric overlap between the planned implant and a segmentation of the acquired implant position [15]. This volumetric overlap measure provides only limited insight in the parameters which are responsible for implant dislocation. The measurements proposed by Stoor et al. are affected by rotational and translational displacement errors, but individual assessment of these parameters is not possible. The innovative method proposed for preformed implants in this article has three major advantages over traditional measurement methods. Firstly, it provides individual measures for all degrees of freedom in implant position; all possible dislocation parameters of the acquired position can be distinguished and quantified. Secondly, the measurements are largely automatic and therefore robust. Segmentation combined with automatic registration prevents emergence of observer-related errors and/or variability. Lastly, the implant position is related to the planned implant position instead of the mirrored orbit. This abolishes possible sources of inter-patient variability or error associated with the mirroring process and assures that outcomes between patients and observers can be compared for all variables.

A disadvantage of the proposed method may be the influence of bending of the implant during surgery. The ICP algorithm searches the best possible fit between the surface of the planned implant model and the segmentation of the implant in the postoperative scan. The accuracy of the match on an intraoperatively bended implant will be hampered by its change in shape in relation to the planned implant. The concept of preformed implants ideally should make bending of the implant obsolete. Using only part of the surface of the implant for matching is possible, to exclude the bended areas and provide the best possible match on the region of interest of the implant. Another drawback of the method could be the choice of the reference axes. A different orientation of the implant’s reference frame will result in differences in the results. Care has been taken to design a logical and intuitive coordinate frame for the implant. It is strongly suggested that the same Orbital Implant Positioning Frame is used for a preformed implant, to be able to compare surgical outcome between different surgeons, methods and studies. Embedding an indication of one or multiple axes of the reference frame in the implant’s design, as well as making the reference implant used in this study available, could aid in standardizing the used reference frame and increasing comparability of studies.

Postoperatively, the analysis method aids in accurate evaluation of surgical result and may assist in choices for re-operations in patients with suboptimal clinical outcome. If intraoperative imaging is performed, the proposed Orbital Implant Positioning Frame can quantify all parameters involved in the obtained implant position. Combined with 3D visualization of the implant dislocation, the Orbital Implant Positioning Frame can provide accurate and intuitive feedback to the surgeon about the necessary adjustments to reposition the implant on the planned position within the same surgical session. This produces a shift in the use of the Positioning Frame: it is not only used to measure position, but it can be used in the positioning process during surgery. The shift to a positioning frame actively providing feedback to the surgeon intraoperatively has even greater implications in combination with intraoperative navigation. The use of intraoperative navigation hardware makes tracking of the implant position within the OR possible. To guide the implant from the current position in the OR to the target position, it is necessary to relate current position to the target position. The Orbital Implant Positioning Frame fulfills this requirement and could thus be used to provide real-time feedback to the surgeon for positioning the implant at the target location.

## Conclusion

This study proposes a novel method of quantifying the outcome of orbital reconstructive surgery. All parameters influencing implant position are quantified, and at the same time the observer-related and patient-related factors are eradicated from the results. Clinically, this method is able to compare different surgical methods for predictability of the reconstruction, provide direct feedback intraoperatively and provide more thorough information about implant position to the surgeon postoperatively. It is also holds a valuable scientific possibility to compare the results in case-series and multi-center observations.

## Supporting Information

S1 Table Resulting implant position analyzed with Orbital Implant Positioning Frame. The rotational parameters (in degrees) and translational parameters (in mm) are given for every specimen.
(XLSX)
